# Child friendly spaces impact across five humanitarian settings: a meta-analysis

**DOI:** 10.1186/s12889-019-6939-2

**Published:** 2019-05-15

**Authors:** Sabrina Hermosilla, Janna Metzler, Kevin Savage, Miriam Musa, Alastair Ager

**Affiliations:** 1 Columbia University, 1051 Riverside Drive, New York, 10032 USA; 2 World Vision International, Chemin de Balexert 7-9, 1219 Châtelaine, Geneva, Switzerland; 3 Institute for Global Health and Development, Queen Margaret University, Edinburgh, Scotland EH21 6UU UK

**Keywords:** Children, Youth, Psychosocial wellbeing, Mental health, Protection, Development, Assets, Resources, Humanitarian, Intervention

## Abstract

**Background:**

Humanitarian crises present major threats to the wellbeing of children. These threats include risks of violence, abduction and abuse, emotional distress and the disruption of development. Humanitarian response efforts frequently address these threats through psychosocial programming. Systematic reviews have demonstrated the weak evidence-base regarding the impact of such interventions. This analysis assesses the impact of Child Friendly Spaces (CFS), one such commonly implemented intervention after humanitarian emergencies.

**Methods:**

We completed baseline and endline (three-six months post-baseline) assessments regarding protection concerns, psychosocial wellbeing, developmental assets and community resources for a total of 1010 children and 1312 carers in catchment areas for interventions with humanitarian populations in Ethiopia, Uganda, Iraq, Jordan, and Nepal. We estimated intervention effect-sizes with Cohen’s *d* for difference in mean difference scores between attenders and non-attenders – who proved comparable on baseline measures – by site. We then pooled findings for a meta-analysis summarizing overall impacts across domains.

**Results:**

Amongst children aged 6–11, significant intervention impacts were observed through site-level analysis for protection concerns (Ethiopia, Cohen’s *d* = 0.48, 95% CI 0.08–0.88), psychosocial wellbeing (Ethiopia, *d* = 0.51, 95% CI 0.10–0.91; and Uganda, *d* = 0.21, 95% CI 0.02–0.40), and developmental assets (Uganda, *d* = 0.37, 95% CI 0.15–0.59; and Iraq, *d* = 0.86, 95% CI 0.18–1.54). Pooled analyses for this age group found impacts of intervention to be significant only for psychosocial wellbeing (*d* = 0.18, 95% CI 0.03–0.33). Among children aged 12–17, site-level analysis indicated intervention impact for protection concerns in one site (Iraq, *d* = 0.58, 95% CI 0.07–1.09), with pooled analysis indicating no significant impacts.

**Conclusion:**

CFS can provide – albeit inconsistently - a protective and promotive environment for younger children. CFS show no impact with older children and in connecting children and carers with wider community resources. A major reappraisal of programming approaches and quality assurance mechanisms is required.

**Electronic supplementary material:**

The online version of this article (10.1186/s12889-019-6939-2) contains supplementary material, which is available to authorized users.

## Background

Humanitarian crises present a broad range of protection risks for children. Conflict and natural disaster expose children to life-threatening events and substantive risks associated with family separation and displacement, including neglect, sexual exploitation and abduction [1]. Exposure to traumatic events and disruption of pre-existing patterns of care bring further challenges to psychosocial wellbeing [2]. Child Friendly Spaces (CFS) have become a standard approach to address the protection and psychosocial needs of children in the context of humanitarian emergencies, being included in several major intervention guidelines [3–5]. CFS are seen as a means of providing a temporary, safe environment in which children may establish some degree of normalcy supportive of their well-being in situations of extreme adversity [5, 6]. CFS are attractive to practitioners wanting a scalable programme with adaptable and diverse activities that is easily deployable in challenging contexts and potentially cost effective [6–11]. CFS, typically established to operate for the first three to six months of a crisis (though potentially extending for longer periods), have recently been documented as part of humanitarian response across the Middle East (including Lebanon, Jordan, Turkey, and Iraq), sub-Saharan Africa (including South Sudan, Central African Republic, Democratic Republic of Congo, Uganda and Cameroon), south-east and south Asia (including the Philippines, India and Bangladesh) and Latin America (including Mexico, Peru and Brazil) [12].

Notwithstanding variation in implementation approach across contexts, the core aims of CFS programming are typically threefold [7, 13]. First, CFS serve as a mechanism for protecting children from abuse, exploitation and violence by providing a safe environment following displacement and/or local disruption. Safety is provided through identification of a secure physical space - repurposed accommodation, tents or a purpose-built construction – and deployment of local adult facilitators to supervise children present. Second, CFS seek to promote children’s psychosocial wellbeing and the resources available to them through structured activities organised by these local facilitators. Whether involving sports, games, songs, educational activities etc. or more specific psychosocial exercises, these seek to build personal resources such as self-esteem and emotional self-regulation as well as broader coping strategies. Third, CFS generally aim to strengthen local mechanisms for support, protection and care for children by serving as a focal point for engaging with parents and mobilising other community resources (including non-governmental providers and relevant civic and religious associations).

Despite widespread consensus on the intervention’s key objectives and subsequent global adoption of its use in emergency settings, little robust evidence exists related to programmatic outcomes and impacts [7, 8]. In a systematic review of published and grey literature, only ten studies were found meeting the inclusion criteria, with most displaying major design weaknesses that restricted the ability to robustly confirm positive change over time or attribute such change to programmatic efforts [7]. Recent broader review of the evidence base for humanitarian interventions addressing the mental health and psychosocial wellbeing of displaced populations echoes these findings, emphasizing the need for more rigorous, mixed methods research on the effectiveness of widely-used, group-based psychosocial interventions, particularly those aimed at children and adolescents, such as CFS [14–17].

## Methods

The current paper draws upon data collected in the course of a series of country field studies completed in a collaboration by Columbia University and World Vision International, working with Save the Children, UNICEF and other members of the Child Protection Working Group of the UN Protection Cluster. By pooling analyses across five sites – spanning Africa, the Middle East and Asia, as well as contexts of both natural disaster and conflict-induced displacement – this paper provides the first robust estimate of the general impact of CFS as a humanitarian intervention.

### Intervention sites

This paper draws on data from five CFS field studies - in Ethiopia, Uganda, Iraq, Jordan, and Nepal (see Table 1) - described in detail elsewhere [18, 19]. Briefly, in the Buramino refugee camp Ethiopia, two CFS sites, with a focus on functional literacy and numeracy skills for children fleeing drought and conflict in Somalia, were evaluated from January–May 2012 [20]. In Uganda, eight CFS were evaluated within the Rwamwanja Resettlement Center, from October 2012–March 2013, where Congolese children escaping conflict primarily engaged in traditional song, dance, storytelling, and organized sports [21]. In the Domiz refugee camp of Iraq, one CFS was evaluated from September 2013 to March 2014, in which Syrian children escaping war engaged in activities such as music, sports, drawing, storytelling, drama, and dance [22]. Similarly, in Zarqa, Jordan, one CFS, which served Syrian children with activities primarily including drawing, handicrafts, puzzles, games, storytelling, singing, and drama, was evaluated from February to August 2014 [23]. In Nepal, 11 CFS serving children affected by the 2015 earthquakes, with activities including games, outdoor sports, creative activities, traditional song and dance, and various life skills were evaluated from March 2015 to May 2016 [24].

**Table 1:** Data collection time period for included studies

Study	Data Collection Wave	
	Baseline (T1)	Endline (T2)
Ethiopia	January 2012	May 2012
Iraq	September 2013	March 2014
Jordan	February 2014	August 2014
Nepal	May 2015	November 2015
Uganda	October 2012	March 2013

Table shows the start time of data collection by site and wave; data collection took on average four to six weeks to complete

### Measures

Measures were identified for four domains (see Table 2). For measures of protection concerns and knowledge of community resources, items were drawn from the Child Protection Rapid Assessment (CPRA, sections one and three respectively), an inter-agency tool designed for use following the rapid-onset of an emergency [25]. For measures of psychosocial wellbeing, a scale with established validity for the target population was identified: the Amharic version of the Strengths and Difficulties Questionnaire [26]; items drawn from a study of child wellbeing in Uganda [27]; an Arabic psychosocial measure developed for use in the Middle East [28]; and versions of the Short Mood and Feelings Questionnaire and Child Hope Scale validated for use in Nepal [29]. Measures of development assets drew upon the SEARCH Institute’s Developmental Assets Profile [30], assessing the presence of internal and external assets supporting development. All measures were translated and back-translated between English and relevant local languages. Acceptable internal consistency was secured across studies for the majority of multi-item scales (Cronbach’s alpha 0.7 or above, see Additional file 1). CFS attendance was established at endline via child or carer report and dichotomized to ‘always’ or ‘frequent’ attender and ‘infrequent’ or ‘never’ attender.

**Table 2:** Data collection tools by study

Study	Data Collection Tools by Respondent Type							
	Child Protection Concerns		Psychosocial Wellbeing		Developmental Assets		Community Resources	
	Caregiver	Child	Caregiver	Child	Caregiver	Child	Caregiver	Child
Ethiopia	CPRA1	CPRA1	SDQ	SDQ	CRDA	B-DAP		
Uganda	CPRA1		CWB		CRDA		CPRA3	
Iraq	CPRA1	CPRA1	MEPS	MEPS	CRDA	EmDAP	CPRA3	CPRA3
Jordan	CPRA1	CPRA1	MEPS	MEPS	CRDA	EmDAP	CPRA3	CPRA3
Nepal	CPRA1	CPRA1	SMFQ	Hope		EmDAP	CPRA3	CPRA3

*CPRA* Child Protection Rapid Assessment (section 1: child protection concerns, or 3: knowledge of community resources to address child protection concerns). *CRDA* Caregiver Rating of Developmental Assets. *CWB* Child Psychosocial Well-being. *DAP* Developmental Assets Profile (Emergency or Brief). *Hope* Child Hope Scale. *MEPS* Middle East Psychosocial Measure. *SDQ* Strengths and Difficulties Questionnaire. *SMFQ* Short Mood and Feelings Questionnaire

### Design

At each site we completed baseline surveys with carers and children from households selected within the catchment area for a proposed CFS before the beginning of programme activities. Procedures to secure equi-probability of selection varied across sites to accommodate local conditions, but generally involved random selection of geographical areas within the catchment area, and then random selection of households within these selected areas [31]. If there was at least one child aged between 6 and 11 in the household, their primary caregiver was identified to be invited for interview with respect to that child (or a randomly selected child in that age range if there was more than one). If there was a child aged between 12 and 17 in the household, that child (or a randomly selected child in that age range if there was more than one) was invited to be interviewed. Informed by local child protection specialists’ judgements of threshold age of consent, ethical approvals required some adjustments in these age cut-offs for self- or caregiver-interview across sites (see Additional file 2). Targeted sample sizes were determined using an established algorithm for detection of an anticipated effect size of between 0.20 and 0.30, with a power of 0.80 and statistical significance of <.05 [32]. For endline assessment, we interviewed these same caregivers and children three to six months later, noting CFS attendance in the intervening period. Strategies to randomly allocate children to attendance and non-attendance conditions proved ethically and pragmatically challenging. Accordingly, comparability of attenders and non-attenders was established by statistical analysis of baseline characteristics. All interviews were completed in the relevant local language(s) by a trained local researcher with fluency in that language. Informed consent to be interviewed was secured verbally, with participants asked to make their ‘mark’ or provide their signature acknowledging willingness to participate. Written copies of the consent form in the relevant local languages were available. The study protocol was reviewed and approved by the Columbia University Medical Center IRB (Reference IRB-AAAJ4352) and relevant national authorities in each setting*.*

### Data analysis

Two authors (SH and MM) extracted relevant information from original CFS study datasets, including child or adolescent’s age, gender, CFS treatment status (intervention or control), and outcome related data (e.g., child protection concerns, psychosocial wellbeing, development assets, and community resources). We assessed the methodological quality of included studies, informed by the PRISMA Statement [33].

We conducted all data analyses in Stata 14. First, to estimate programme effect, we calculated crude Cohen’s *d* for each study site across all primary outcomes (recoded when necessary so a positive outcome is indicative of a salubrious programme effect), then stratified by age (under 12 and 12 and over age categories were selected across all study sites to be consistent with onset of adolescents in the literature, independent of respondent type) and gender. A Cohen’s *d* of 0.20 is considered a small effect, 0.50 a moderate effect, and 0.80 or above, a large effect [34]. Second, to estimate the effect across all sites, we conducted a random-effects meta-analysis with bootstrapped DerSimonian-Laird variance estimation for all primary outcomes and age and gender stratifications [35].

As CFS site inclusion was determined based on intervention rigor, from internal study documents rather than a published literature search, we did not conduct publication bias tests. We assessed heterogeneity across sites with both the χ^2^ test for heterogeneity (statistical significance at 0.05) and *I*^2^ (50% indicative of moderate heterogeneity).

### Role of the funding source

The funders had no role in the design or conduct of data analyses, nor in interpretation of findings. SH, MM, JM, and AA had full access to all data and bear responsibility for publication.

## Results

Across the five CFS field studies (Ethiopia, Uganda, Iraq, Jordan, and Nepal) we extracted baseline and endline (three to six months post-baseline) data on 1010 children and 1312 carers. Studied children ranged in age from six to seventeen. At baseline there were few statistically significant differences in age, gender, or outcome measures between CFS attenders and non-attenders (Table 3), supporting the validity of the latter as a counterfactual control condition. CFS programming across sites differed for younger and older children, thus we report age-stratified (under twelve years and twelve years and over) meta-analytic results. Sample size constraints prevented gender-stratified analyses from converging across more than two studies, and are thus not presented.

**Table 3:** Baseline sample characteristics by study

	Ethiopia		Uganda		Iraq		Jordan		Nepal	
	Attenders	Non-Attenders	Attenders	Non-Attenders	Attenders	Non-Attenders	Attenders	Non-Attenders	Attenders	Non-Attenders
	Mean (SD)	Mean (SD)	Mean (SD)	Mean (SD)	Mean (SD)	Mean (SD)	Mean (SD)	Mean (SD)	Mean (SD)	Mean (SD)
Crisis Context	Natural Disaster, Conflict		Conflict		Conflict		Conflict		Natural Disaster	
Age	10.97 (2.87)	11.28 (2.86)	9.36^*^ (2.00)	8.06^*^ (1.85)	9.57^*^ (2.15)	11.07^*^ (3.05)	10.53 (3.03)	10.84 (3.04)	9.93 (9.68)	9.96 (2.46)
Gender, female, n (%)	79.00 (45.40)	45.00 (47.87)	76.00 (44.71)	247.00 (53.35)	36.00 (52.17)	64.00 (43.24)	155.00 (53.45)	60.00 (56.07)	161.00 (48.49)	226.00 (47.58)
Protection concerns	0.06 (0.24)	0.12 (0.03)	5.55 (1.93)	5.46 (2.04)	2.41 (1.51)	2.18 (1.64)	3.18 (2.87)	3.02 (3.15)	2.97 (2.17)	2.97 (2.20)
Psychosocial wellbeing	16.77 (3.35)	17.67 (3.81)	12.33 (5.03)	12.27 (4.15)	8.38^*^ (3.11)	7.62^*^ (3.21)	8.85 (4.19)	9.21 (4.50)	12.23^*^ (4.69)	11.98^*^ (4.34)
Development assets	17.43 (4.84)	18.37 (5.39)	14.00^*^ (5.39)	15.20^*^ (5.45)	21.02 (5.28)	19.99 (4.07)	25.93^*^ (6.75)	27.57^*^ (6.84)	27.00 (6.21)	26.72 (5.81)
Community resources knowledge	–	–	0.65 (1.16)	0.50 (0.96)	0.57 (0.61)	0.44 (0.56)	2.26 (2.09)	2.06 (2.06)	1.62 (1.21)	1.68 (1.31)

Notes. Community resources knowledge was not asked about in Ethiopia. *Indicates statistically significant difference (*p* < 0.05) between subsequent attenders or non-attenders of CFS at baseline

### Protection concerns

**Fig. 1:**
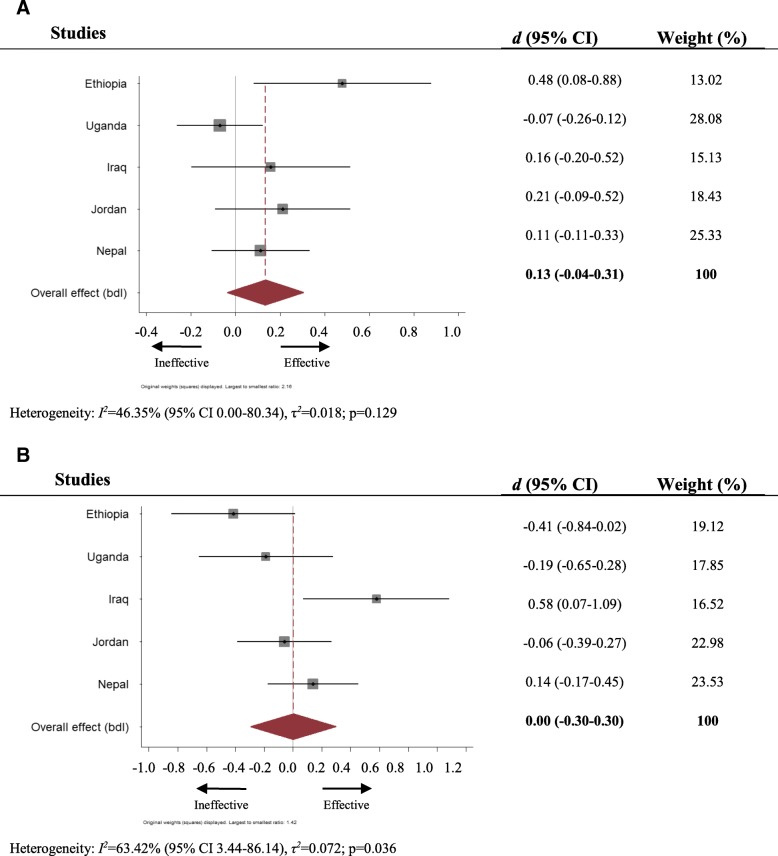
**a**. Forest plot of effect of CFS attendance on child protection concerns, children under 12 years old. **b**: Forest plot of effect of CFS attendance on child protection concerns children 12 years old and older

In four of the five studies (see Fig. 1), CFS attendance was associated with a reduced reporting of protection concerns for caregivers of children aged 6–11, although only in the case of Ethiopia was this trend statistically significant (Cohen’s *d* = 0.48, 95% CI 0.08–0.88). Pooled analysis suggested an overall weak, and statistically insignificant, intervention effect size of 0.13 (− 0.04, 0.31, *I*^2^ = 46.35%) for this age group. For children aged twelve and older, the pooled Cohen’s *d* was 0.00 (− 0.30, 0.30) and marked by significant heterogeneity (*I*^*2*^ = 63.42%, *p* < .036). Only in Iraq was there a significant trend for reduced reporting of protection concerns amongst CFS attenders amongst this age group (Cohen’s *d* = 0.58, CI 0.07–1.09).

### Psychosocial wellbeing

**Fig. 2:**
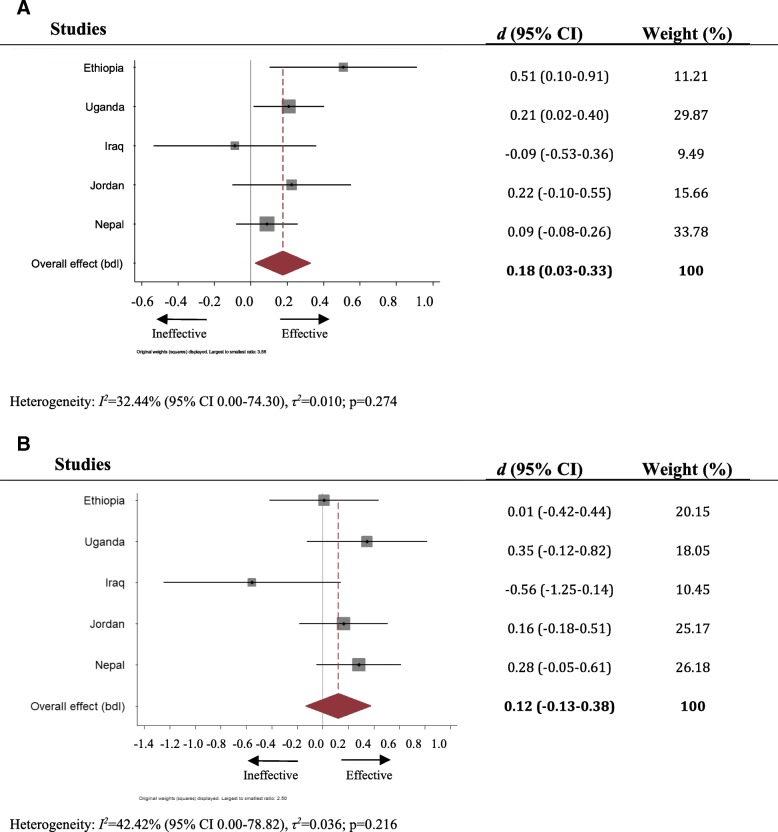
**a**. Forest plot of effect of CFS attendance on psychosocial wellbeing, children under 12 years old. **b**: Forest plot of effect of CFS attendance on psychosocial wellbeing, children 12 years old and older

In four of the five studies (see Fig. 2), CFS attendance was associated with reports of better wellbeing amongst children aged 6–11. This trend was statistically significant in Ethiopia (Cohen’s *d* = 0.51, 95% CI 0.10–0.91) and in Uganda (Cohen’s *d* = 0.21, 95% CI 0.02–0.40). Pooled analysis suggested a small net intervention effect (Cohen’s *d* = 0.18, 95% CI 0.03–0.33**)** for this age group. For children aged twelve and older, the pooled Cohen’s *d* was 0.12 (− 0.13, 0.38), with no significant trend for better outcomes regarding psychosocial wellbeing amongst CFS attenders indicated at any site.

### Developmental assets

**Fig. 3:**
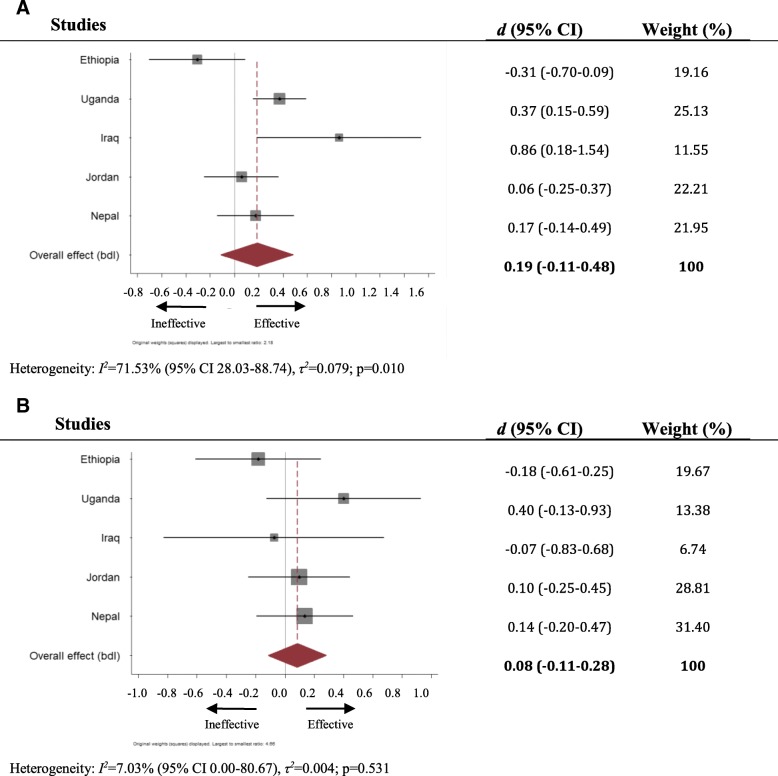
**a** Forest plot of effect of CFS attendance on developmental assets, children under 12 years old. **b**: Forest plot of effect of CFS attendance on developmental assets, children 12 years old and older

In four of the five study sites, amongst children aged 6–11 greater positive impact on developmental assets was observed for those who had attended a CFS (see Fig. 3). This trend was statistically significant – with moderate to large effect size - in both Uganda (Cohen’s *d* = 0.37, 95% CI 0.15–0.59) and Iraq (Cohen’s *d* = 0.86, 95% CI 0.18–1.54). Pooled analysis suggested an overall weak, statistically insignificant, and highly heterogeneous intervention effect of 0.19 (− 0.11, 0.48, *I*^*2*^ = 71.53%, *p* < .010) for this age group,

For older children, the pooled Cohen’s *d* was 0.08 (− 0.11, 0.28), with no significant trend for better outcomes regarding developmental assets amongst CFS attenders indicated at any site.

### Knowledge of community resources

**Fig. 4:**
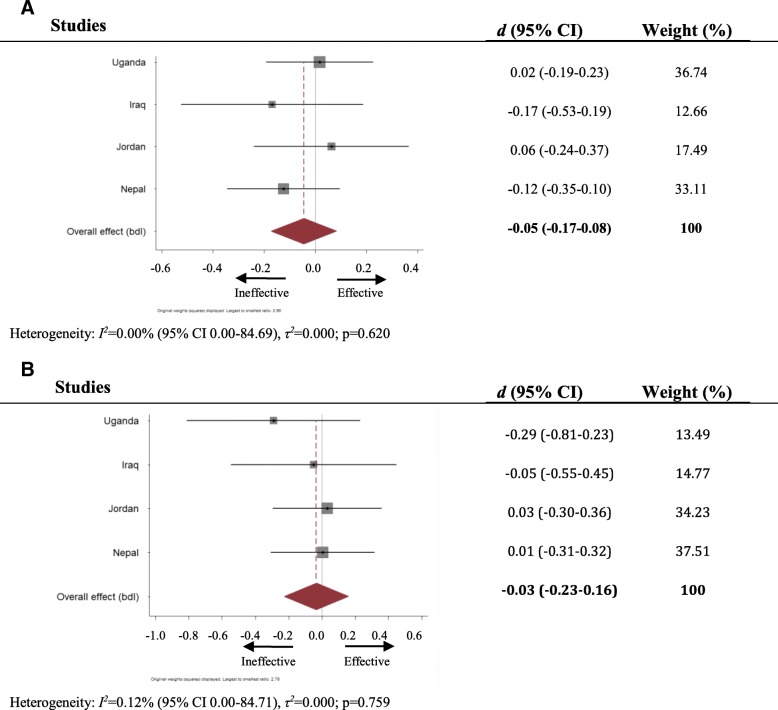
**a** Forest plot of effect of CFS attendance on knowledge of community resources, children under 12 years old. **b**: Forest plot of effect of CFS attendance on knowledge of community resources, children 12 years old and older

Across Uganda, Iraq, Jordan, and Nepal (items were not used in first study in Ethiopia), there was no site where CFS attendance predicted greater knowledge of community resources (see Fig. 4). Pooled analysis accordingly indicated no statistically significant effect of CFS attendance on such knowledge for either age group (under 12: Cohen’s *d* − 0.05, 95% CI: − 0.17-0.08; 12^+^ Cohen’s *d* − 0.03, 95% CI: − 0.23-0.16).

## Discussion

Our evidence across five field settings suggests that CFS can effectively address protection risks, threats to psychosocial wellbeing and support developmental assets amongst younger children. However, the extent to which targeted protective and promotive impacts are achieved by CFS varies widely across domains and settings.

The largest and most consistent evidence of impact is in relation to psychosocial wellbeing and developmental assets. The effect sizes on these measures, 0.18 and 0.19 respectively, represent modest but - with the large potential population influenced – substantive change of clear humanitarian consequence in terms of the mandate to reduce suffering and promote dignity [36–39]. As noted, however, these pooled effect sizes reflect significant heterogeneity across settings. If the largest impacts identified were more consistently achieved this would transform CFS into an intervention with a moderate to high effect size. Establishing the basis for variation in outcomes is clearly a key priority for future systematic study. However, from field reports it appears likely that the orientation of activities, the quality assurance of programming and the influence of the site setting all play a potential role [18–24].

Impacts of CFS on child protection for younger children are overall more modest (with a pooled effect size of 0.13) but again important in humanitarian settings commonly marked by insecurity and with large populations of children [39]. Heterogeneity of impacts here is plausibly linked to the characteristics of programming and context. For example, the CFS in Ethiopia (where the largest effect size of 0.48 was observed) secured sites with chain fences and employed security guards to separate children from external risks while attending; the CFS in Uganda (where the weakest effect was observed) were open sites less clearly demarked within the settlement [20, 21]. Children and carers also identified some protection concerns in the wider environment over which CFS would feasibly have modest influence.

In the domain of knowledge of community resources there was no evidence of CFS impact on younger – nor older – children. With the potential for the benefits of knowledge gained through CFS (e.g. regarding organizations providing assistance or how to report protection concerns) to be shared within communities to non-attending households, it is possible that our research design was insensitive to broader community awareness established through CFS. However, with the non-significant trend actually towards greater knowledge amongst non-attenders than attenders there are weak grounds for assuming CFS to be a driver of such awareness.

Other than a small pooled effect (0.12) with regarding to psychosocial wellbeing, we found no evidence of CFS being an impactful intervention for older children across any domain. Engaging youth is recognised as challenging in humanitarian contexts given pressures on household livelihoods. However, Panter-Brick and colleagues [39] have recently documented moderate impacts (Cohen’s d between 0.30 and 0.40) on insecurity, distress and well being of an eight-week profound stress atunement framework-informed programme of structured activities. This suggests that intervention approaches need to be more explicitly shaped to the interests and circumstances of youth if they are to be impactful in humanitarian settings.

Overall, given the dearth of rigorous evaluations of psychosocial interventions in emergency settings [17, 40] and the relatively wide-scale use of CFS, these findings provide valuable insight into the role that CFS are playing in the lives of tens of thousands of (younger) children and their families in the context of humanitarian emergencies. Critically, however, our study demonstrates that the impact of the intervention varies widely and is frequently below ‘best in class’ effectiveness. Research is required to elucidate the drivers of this variation. Field reports suggest that programme content, adaptation to context, and adherence to quality standards are likely key issues to focus upon [18]. For instance, the potential benefits of programming priorities addressing specific local circumstances, such as peace-building activities being incorporated into CFS in conflict-affected contexts or community mapping of earthquake ‘safe’ zones in post-earthquake settings, warrants exploration. Recently developed agency guidance for CFS in humanitarian settings [41], which has drawn upon the research studies reported here, posits an adequate supervisor-to-child ratio, attendance verification, forethought in activity planning and implementation, and a well-maintained safe play environment for children as hallmarks of quality CFS provision. Attention also needs to be paid to strategies to strengthen engagement of CFS - and other programming approaches – with community resources in a way that will not only bolster intervention impact but also provide a sustainable basis for supporting longer-term impacts. This will require consideration of models of resource mobilization that foster localisation of humanitarian response from the earliest stages of an emergency. There is potential for such studies of comparative approaches to CFS to adopt cluster randomized control, or stepped wedge, designs that were not achievable for the current attender vs. non-attender analyses.

## Conclusions

CFS can effectively address protection risks and threats to psychosocial wellbeing and support developmental assets amongst younger children. Effect sizes are modest but potentially valuable at the population level given the typically wide scale of implementation. However, these impacts are not consistently observed, suggesting the need for strengthened contextual adaptation and quality control and monitoring systems of implementing agencies. Impacts with older children, and on mobilization of community resources to support children, are notably weak, suggesting the need to consider alternative programming approaches if gains in these areas are to be secured.

## Additional files

Additional file 1: Internal Consistency of Scales at Baseline by Respondent Type. (DOCX 23 kb)

Additional file 2: Age and Sample Size at Baseline by Respondent Type. (DOCX 23 kb)

## Abbreviations

CFS: Child Friendly Spaces
CPRA: Child Protection Rapid Assessment
