# Supplementary material for: Child friendly spaces impact across five humanitarian settings: a meta-analysis
Source: BMC Public Health. 2019 May 15;19:576. doi: 10.1186/s12889-019-6939-2 (PMC6521445; doi:10.1186/s12889-019-6939-2)
Supplement: Supplementary file 1 — Internal Consistency of Scales at Baseline by Respondent Type. (DOCX 23 kb) [file 12889_2019_6939_MOESM1_ESM.docx]

|  | **Psychosocial Wellbeing** | | | **Developmental Assets** | |
| --- | --- | --- | --- | --- | --- |
|  | **Caregiver** | **Child** | **Caregiver** | | **Child** |
| Ethiopia | SDQ (0.79) | SDQ (0.76) | CRDA (0.62) | | B-DAP (0.71) |
| Uganda | CWB (0.66) | - | CRDA (0.79) | | - |
| Iraq | MEPS (0.63) | MEPS (0.63) | CRDA (0.59) | | EmDAP (0.59) |
| Jordan | MEPS (0.86) | MEPS (0.86) | CRDA (0.78) | | EmDAP (0.75) |
| Nepal | SMFQ (0.84) | Hope (0.71) | - | | EmDAP (0.79) |

**Additional File A. Internal Consistency of Scales at Baseline by Respondent Type**

CRDA= Caregiver Rating of Developmental Assets. CWB= Child Psychosocial Well-being. DAP= Developmental Assets Profile (Emergency or Brief). Hope=Child Hope Scale. MEPS= Middle East Psychosocial Measure. SDQ= Strengths and Difficulties Questionnaire. SMFQ=Short Mood and Feelings Questionnaire.
