# Supplementary material for: Child friendly spaces impact across five humanitarian settings: a meta-analysis
Source: BMC Public Health. 2019 May 15;19:576. doi: 10.1186/s12889-019-6939-2 (PMC6521445; doi:10.1186/s12889-019-6939-2)
Supplement: Supplementary file 2 — Age and Sample Size at Baseline by Respondent Type. (DOCX 23 kb) [file 12889_2019_6939_MOESM2_ESM.docx]

| **Study** | | **Caregiver** | | **Child** | | **Totals** |
| --- | --- | --- | --- | --- | --- | --- |
|  |  | **Attender** | **Non-Attender** | **Attender** | **Non-Attender** |  |
| Ethiopia | age | 6 – 11 years old | | 12 – 17 years old | | 6 – 17 years old |
|  | n | 100 | 43 | 74 | 51 | 268 |
| Uganda | age | 6 – 12 years old | | - | | 6 – 12 years old |
|  | n | 170 | 463 |  |  | 633 |
| Iraq | age | 7 – 11 years old | | 12 – 16 years old | | 7 – 16 years old |
|  | n | 49 | 79 | 20 | 69 | 217 |
| Jordan | age | 6 – 9 years old | | 10 – 18 years old | | 6 – 18 years old |
|  | n | 128 | 37 | 162 | 70 | 397 |
| Nepal | age | 6 – 8 years old | | 9 – 17 years old | | 6 – 17 years old |
|  | n | 107 | 136 | 225 | 339 | 807 |
| **Study Total** | n | 554 | 758 | 481 | 529 | 2,322 |

**Additional File B. Age and Sample Size at Baseline by Respondent Type**

Note. No child (12-17) reports for Uganda.
